# Advanced glycation end products and their soluble receptor (sRAGE) in patients with Hashimoto’s thyroiditis on levothyroxine substitution

**DOI:** 10.3389/fendo.2023.1187725

**Published:** 2023-05-26

**Authors:** Sára Csiha, István Molnár, Sándor Halmi, Dávid Hutkai, Hajnalka Lőrincz, Sándor Somodi, Mónika Katkó, Mariann Harangi, György Paragh, Endre V. Nagy, Eszter Berta, Miklós Bodor

**Affiliations:** ^1^ Division of Endocrinology, Department of Medicine, Faculty of Medicine, University of Debrecen, Debrecen, Hungary; ^2^ Department of Clinical Basics, Faculty of Pharmacy, University of Debrecen, Debrecen, Hungary; ^3^ Doctoral School of Health Sciences, University of Debrecen, Debrecen, Hungary; ^4^ Division of Metabolism, Department of Medicine, Faculty of Medicine, University of Debrecen, Debrecen, Hungary; ^5^ Division of Nephrology, Department of Medicine, Faculty of Medicine, University of Debrecen, Debrecen, Hungary; ^6^ Kálmán Laki Doctoral School, University of Debrecen, Debrecen, Hungary; ^7^ Department of Emergency Medicine, Faculty of Medicine, University of Debrecen, Debrecen, Hungary; ^8^ Institute of Health Studies, Faculty of Health Sciences, University of Debrecen, Debrecen, Hungary

**Keywords:** thyroid, advanced glycation end products, sRAGE, hypothyroidism, Hashimoto’s thyroiditis, levothyroxine, hyperlipidemia, obesity

## Abstract

**Background:**

Advanced glycation end products (AGEs) are heterogenous group of irreversible chemical moieties originated from non-enzymatic glycation and oxidation of proteins, nucleic acids, and lipids. The engagement of AGEs with their chief cellular receptor (RAGE) activates a myriad of signaling pathways contributing to the progression of chronic diseases like autoimmune thyroiditis, type 2 diabetes mellitus and its complications. Soluble RAGE (sRAGE) prevents AGE-RAGE interaction in a competitive manner.

**Objective:**

We investigated the association between serum AGE, sRAGE and thyroid function in 73 Hashimoto thyroiditis patients (HT) on levothyroxine substitution, and in 83 age, BMI and gender-matched healthy controls.

**Methods:**

The serum AGEs levels were determined by autofluorescence on a multi-mode microplate reader, and the serum sRAGE levels by ELISA method.

**Results:**

Mean AGE level was lower (10.71 vs 11.45 AU/µg protein; p=0.046), while mean sRAGE level was higher (923 vs 755 pg/mL; p<0.0005) in the serum of HT patients than the controls. AGE correlated with age, while sRAGE correlated negatively with BMI in both groups. We found negative correlation between AGE and fT3 levels (r=-0.32; p=0.006) and sRAGE and TSH levels (r=-0.27; p=0.022) in HT patients, while we failed to find association between AGE, sRAGE and parameters of thyroid function in the control group. Median AGE/sRAGE ratio was lower in HT patients than in controls (2.4, IQR 1.9 – 3.1 vs 3.3, IQR 2.3 – 4.1 AU/pg; p < 0.001). In HT patients, the AGE/sRAGE ratio correlated positively with BMI and correlated negatively with fT3.

**Conclusion:**

According to our results in HT patients lower TSH and higher fT3 levels within the reference range is accompanied by a favorable AGE/RAGE balance. Further investigations are needed to confirm these results.

## 1 Introduction

Hashimoto’s thyroiditis (HT) is a frequent form of autoimmune thyroid disease with an incidence of 0.3-0.5 cases per 1000 people, being the leading cause of hypothyroidism in iodine sufficient regions of the world (1, 2). Environmental factors and genetic susceptibility together have a fundamental role in the pathogenesis of HT, while advancing age and female gender are also important predisposing factors, the latter carrying an 8-10-fold risk. Several existential and environmental factors such as stress, excessive iodine or inadequate dietary selenium intake, multiparity and viral infections promote the occurrence of HT, while smoking and alcohol consumption seem to have a protective effect (1). Besides thyroid autoimmunity other autoimmune disorders may develop among Hashimoto’s thyroiditis patients such as Sjögren’s syndrome, myasthenia gravis, pernicious anemia, connective tissue disorders, autoimmune liver disease, vitiligo and celiac disease; certain concomitant endocrinopathies of autoimmune origin like type 1 diabetes mellitus, hypoparathyroidism and Addison’s disease together with HT form autoimmune polyendocrine syndromes (2).

In the pathogenesis of Hashimoto’s thyroiditis, increased oxidative stress and decreased antioxidant levels were previously observed (3, 4). In the long term, the disturbed redox balance promotes the generation of endogenously produced Advanced Glycation End-Products (AGEs). In a previous study, a decrease in sRAGE and an increase in AGE levels were confirmed in 50 newly diagnosed euthyroid HT patients, but this relationship has not been investigated yet in HT patient group on stable levothyroxine hormone substitution (5). In an earlier study, Giannakou et al. found the oxidative parameters of LT4-treated patients higher than patients without supplementation need, however, AGE and sRAGE results were not evaluated (6).

In addition to the documented obesogenic effect of hypothyroidism (7), according to growing data obesity is also a non-conventional risk factor for Hashimoto’s thyroiditis and hypothyroidism (8). In a recent publication euthyroid non-obese HT patients’ dietary habits were investigated, and their animal originated food consumption was higher, while plant food intake was lower than the control subjects’ accompanied by higher AGEs level among HT patients (9).

AGEs are a heterogeneous group of irreversible adducts resulting from non-enzymatic glycation and oxidation of proteins, nucleic acids and lipids (10, 11). They can be of exogen and endogen origin and have a slow elimination from the bloodstream, therefore are capable of lifelong accumulation (12, 13). AGEs from exogenous sources also called dietary AGEs are found in large quantities in processed, high-temperature, grilled foods containing a high level of fat and protein (14). Besides food, smoking is an important exogen source of AGEs. AGEs play fundamental role in aging and in the development of various diseases and their complications, including metabolic syndrome, type 2 diabetes mellitus, cancer, atherosclerosis and autoimmune thyroid disease. Furthermore, AGEs and their receptors have significant influence on carcinogenesis and progression of thyroid carcinoma, which with an increasing incidence represents an important public health concern; the growing incidence might be partially attributable to environmental factors like obesity (13, 15).

AGEs have several membrane-bound, cell surface receptors which, following interaction can initiate various signal transduction pathways. Some receptors, such as the advanced glycation end product receptor 1, help eliminate AGEs from the body, others, like RAGE by activating the nuclear factor kappa B signaling pathway increase receptor expression, the release of inflammatory cytokines, oxidative stress, and reactive oxygen species (ROS) formation. The increased oxidative stress can also lead to formation of endogenous AGEs. This phenomenon leads to the development of a self-exciting process, which plays a long-term role in the formation of the above-mentioned chronic diseases and the development of their complications. Furthermore, AGEs can form cross-links with proteins, thus changing their function, adding to the pathogenesis for the wide range of AGE-related diseases (12, 14).

The best characterized AGE receptor is RAGE. It is a member of the superfamily of cell surface immunoglobulins (6) type 1 transmembrane protein (with an N-terminal extracellular domain) with size of 45 kDa and 404 amino acids, originally isolated from bovine pulmonary endothelium (16). In addition to the complete RAGE receptor, several naturally occurring RAGE variants have also been described, like DN-RAGE, ΔN-RAGE and sRAGE (es RAGE and cRAGE). Soluble AGE receptor (sRAGE) competitively prevents AGE-RAGE interaction, thereby reducing the generation of inflammatory cytokines and ROS (11, 14, 17). Decreased serum levels of sRAGE was detected in patients with essential hypertension (18), coronary artery diseases, chronic obstructive pulmonary disease, hyperthyroidism, rheumatic arthritis, and Alzheimer’s disease (19).

A recent work of Prasad summarized the diseases, in which the elevated ratio of AGE/sRAGE was proved to be a biomarker among a wide variety of patients treated for non-ST-elevation myocardial infarction (11), hyperthyroidism (20), thoracic aortic aneurysm and hypercholesterolemia (19, 21).

We aimed to assess the AGE and sRAGE levels, and the AGE/sRAGE ratio and their correlations with thyroid function and metabolic parameters in HT patients on levothyroxine substitution treatment and in age, body mass index (BMI), and gender-matched control subjects. We hypothesized that in HT patients AGE level is higher and sRAGE level is lower than in healthy controls. We supposed correlations between the levels of AGE and parameters of thyroid function and metabolic parameters.

## 2 Materials and subjects

### 2.1 Study population

We investigated the association between serum AGE, serum sRAGE and thyroid function parameters in patients receiving levothyroxine substitution treatment for HT-caused hypothyroidism. Seventy-three Caucasian subjects (69 women and 4 men, mean age: 47 ± 14 years, mean BMI: 27.4 ± 5.6 kg/m^2^, mean duration of the disease 6.6 ± 4.4 years) and eighty-two Caucasian controls (n=82, 76 women and 6 men, mean age: 46 ± 14 years, mean BMI: 27.2 ± 6.1 kg/m^2^) were enrolled from the outpatient clinic of our Endocrine Unit. Expectant mothers and patients with known diabetes mellitus, cancer, or concomitant other autoimmune diseases were excluded. The control group consisted of age, sex, and BMI-matched controls without thyroid disease in their medical history, whom TSH, fT4 and fT3 and TPOAb levels were in the reference range. The study was approved by the Regional and Institutional Ethics Committee of the University of Debrecen. All patients and controls consented in participation and signed the Informed Consent Form.

### 2.2 Sample collection and laboratory measurements

Blood samples were collected according to local clinical protocol. The plasma and serum were separated by centrifugation at 2200 g for 10 minutes, aliquoted and stored at -80°C until later measurements.

The serum AGEs levels were determined by autofluorescence on a multi-mode microplate reader (Biotek Synergy H1, Agilent Technologies, Santa Clara, CA, USA), and the sRAGE levels by ELISA method (Human RAGE Quantikine ELISA Kit, R&D Systems, Minneapolis, MN, USA) according to the manufacturer’s instructions.

The serum free thyroxine (fT4) and free triiodothyronine (fT3) and thyroid stimulating hormone (TSH) levels were measured using electrochemiluminescence immunoassays (FT4 G2 Elecsys, FT3 Elecsys, TSH Elecsys, Roche Diagnostics GmbH, Mannheim, Germany). Reference ranges 12–22 pmol/L, 2.4–6.3 pmol/L and 0.3–4.2 mU/L for fT4, fT3 and TSH, respectively. Anti-thyroperoxidase (aTPO) antibody concentrations were measured by chemiluminescent immunoassay (LIAISON^®^‐Anti-TPO, DiaSorin S.p.A., Saluggia, Italy).

Serum creatinine was measured by Jaffé’s colorimetric method, C-reactive protein (CRP) was measured by immunoturbidimetric assay. Triglyceride, total cholesterol, low-density lipoprotein cholesterol (LDL-C) and high-density lipoprotein cholesterol (HDL-C) were measured using enzymatic, colorimetric tests, and glucose was measured by hexokinase kinetic enzymatic assay with a Cobas c600 autoanalyzer (Roche Diagnostics GmbH, Mannheim, Germany).

### 2.3 Statistical analysis

Statistical analysis was performed by STATISTICA (Statsoft Inc. Tulsa, OK, USA) and Graphpad Prism (Graphpad Software, San Diego, CA, USA). The distribution of continuous variables was checked by the Kolmogorov–Smirnov test. To compare continuous variables between groups, for normal distributed data Student’s t test was applied, whereas for nonnormal distributed data Mann Whitney‐U test was used. Results were expressed as mean ± standard deviation (SD) in case of normal distribution, or median and 25th and 75th percentiles (interquartile range, IQR) in case of nonnormal distribution. The stochastic relationships of discrete variables were analyzed by Chi‐square test. For analysis of the relationship between continuous variables Pearson’s correlation was performed. Multiple linear regression analysis was performed to analyze the relationship between AGEs or sRAGE level as dependent variable and several independent variables. p values below 0.05 were considered statistically significant.

## 3 Results

**Figure 1:**
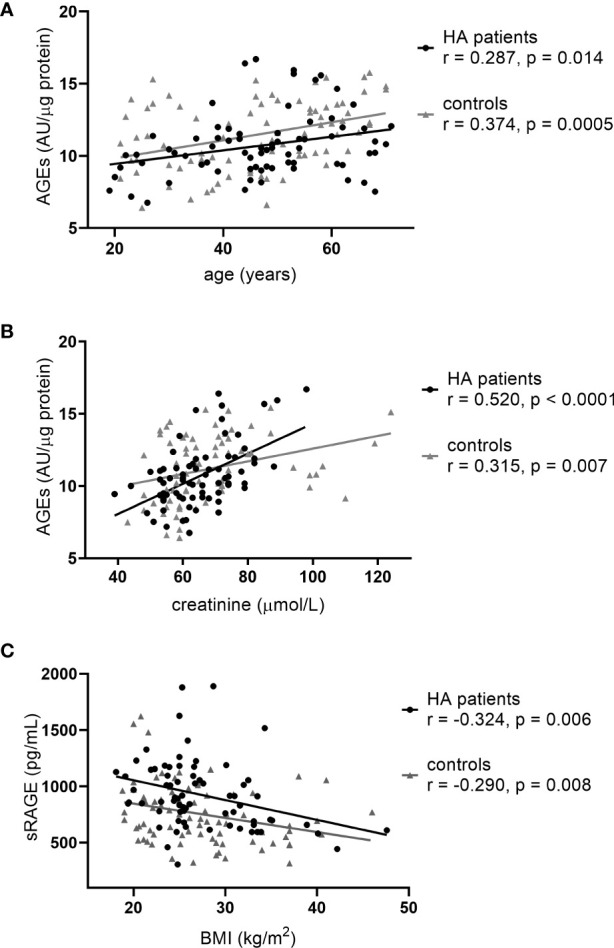
Correlations characteristic to the whole study population: **(A)** age vs AGEs, **(B)** serum creatinine vs AGEs and **(C)** BMI vs sRAGE.

All patients needed levothyroxine (LT4) supplementation with 1.15 µg/kg body weight median prescribed daily dose (IQR: 0.83 – 1.41, minimum-maximum: 0.32 – 3.28), 64% of them were euthyroid at the time of study, subclinical hypo- and hyperthyroidism occurred in 22 and 6% of the cases, while overt hypo- and hyperthyroidism occurred in 4 and 4% of the cases, respectively. The median TSH and fT4 levels of the HT patients were higher (2.47 vs 1.77 mU/L, p=0.024 and 17.6 vs 15.5 pmol/L, p<0.0001, respectively), while mean fT3 level was lower compared to the control group (4.6 vs 5.0 pmol/L, p<0.0001) (**Table 1**). Furthermore, the mean AGE was lower (10.71 vs 11.45 AU/µg protein, p=0.023) while the sRAGE level was higher (923 vs 755 pg/mL, p<0.0005) in the serum of HT patients compared to the controls (**Table 1**). Moreover, AGE was positively correlated with age and serum creatinine (**Figures 1A, B**), whereas sRAGE was negatively correlated with BMI (**Figure 1C**) both in the whole studied population and in subgroups (controls and HT patients) separately. No correlation was found between AGEs and sRAGE levels (r=0.191, p=0.086 in controls; r=0.205, p=0.083 in HT patients and r=0.140, p=0.082 in the whole population).

**Table 1:** Clinical characteristics and laboratory parameters of HT patients and controls.

	HT patients n=73	Controls n=82	p*
Age (years)	47 ± 14	46 ± 14	0.635
Female/Male	69/4	76/6	0.448
BMI (kg/m^2^)	27.4 ± 5.6	27.2 ± 6.1	0.864
TSH (mU/L)	2.47 (0.97 – 4.28)	1.77 (1.25 – 2.38)	0.024
fT4 (pmol/L)	17.6 (15.5 – 20.6)	15.5 (14.2 – 16.5)	<0.0001
fT3 (pmol/L)	4.6 ± 0.6	5.0 ± 0.6	<0.0001
Random glucose (mmol/L)	5.2 (4.8 – 5.6)	5.0 (4.7 – 5.4)	0.082
Triglyceride (mmol/L)	1.4 (1.0 – 2.0)	1.2 (0.8 – 1.7)	0.048
Cholesterol (mmol/L)	5.3 ± 1.0	5.4 ± 1.1	0.597
LDL-C (mmol/L)	3.3 ± 0.9	3.4 ± 1.0	0.525
HDL-C (mmol/L)	1.5 (1.3 – 1.8)	1.5 (1.2 – 1.7)	0.622
CRP (mg/L)	1.7 (0.9 – 3.6)	2.6 (0.9 – 5.5)	0.095
Creatinine (µmol/L)	65 ± 11	69 ± 16	0.160
AGE (AU/µg protein)	10.71 ± 2.21	11.45 ± 2.35	0.046
sRAGE (pg/mL)	923 ± 303	755 ± 265	<0.0005
Median AGE/sRAGE ratio (AU/pg)	2.4 IQR 1.9 – 3.1	3.3 IQR 2.3 – 4.1	p < 0.0001

Data are shown as mean ± standard deviation or median (interquartile range).

* t-test for comparing age, BMI, fT3, AGEs and sRAGE, Chi-square test for comparing female/male ratio and Mann-Whitney U test for comparing other parameters.

**Figure 2:**
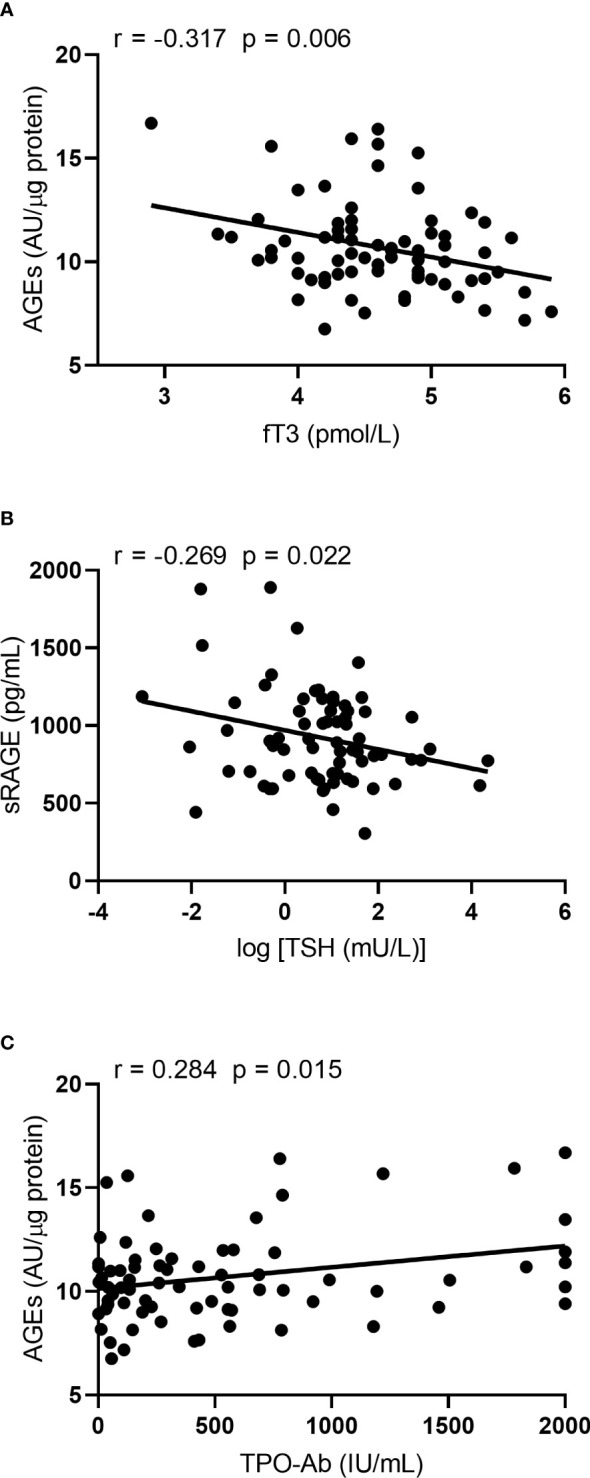
Correlations characteristic to the HT patients: **(A)** fT3 vs AGEs, **(B)** TSH vs sRAGE and **(C)** TPOAb vs AGEs.

**Figure 3:**
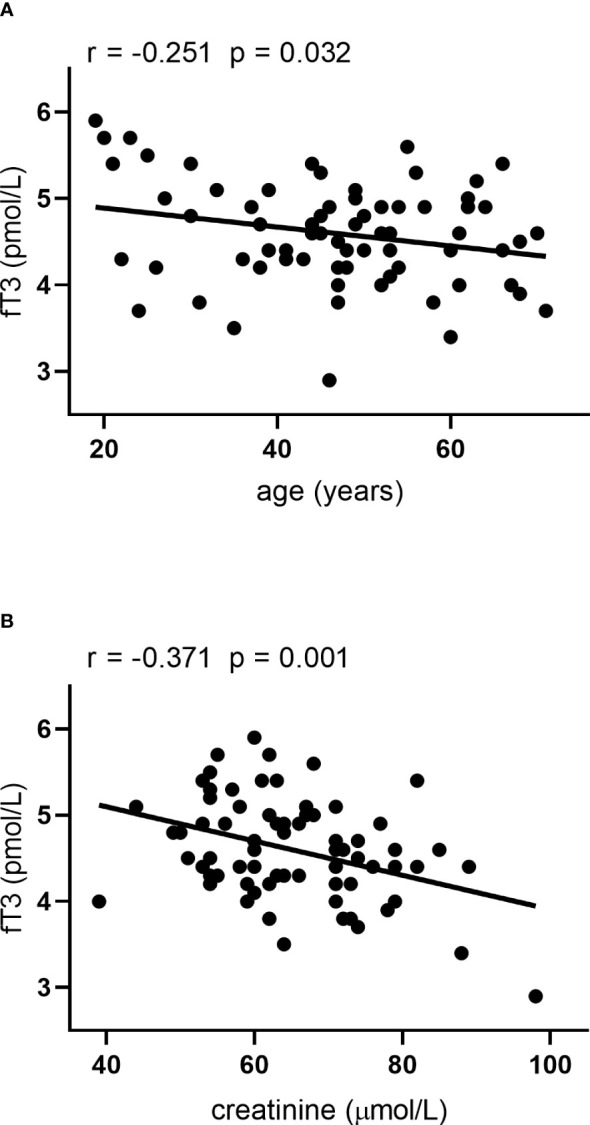
Correlations between fT3 and **(A)** age and **(B)** serum creatinine in HT patients.

A negative correlation was found between the serum AGE concentration and fT3 level (**Figure 2A**), as well as between sRAGE and TSH levels (**Figure 2B**) in HT patients, while in the control group neither AGE nor sRAGE correlated with the parameters of thyroid function (data not shown). In addition, we found positive correlation between TPOAb and AGE level in HT patients (**Figure 2C**). In HT patients fT3 levels correlated with age and serum creatinine levels (**Figure 3**). Since the serum AGE level was correlated with age, creatinine, TPOAb and fT3 level among patients, we performed a multiple regression analysis, in which creatinine and TPOAb levels proved to be significant predictors (**Table 2**). In the case of sRAGE, the same was examined in relation to BMI and TSH, and the effect of both parameters was found to be significant in the multiple regression analysis (**Table 2**).

**Table 2:** Results of the multiple regression analysis.

	β	SE of β	b	SE of b	p
Dependent variable: AGEs					
age	0.187	0.101	0.031	0.017	0.069
se creatinine	0.405	0.107	0.081	0.021	0.0003
TPOAb	0.224	0.099	0.001	0.0004	0.026
fT3	-0.108	0.106	-0.405	0.398	0.313
Dependent variable: sRAGE					
BMI	-0.379	0.108	-20.590	5.865	0.0008
log TSH	-0.353	0.108	-81.435	24.925	0.002

The median AGE/sRAGE ratio was lower in HT patients than in controls (2.4, IQR 1.9 – 3.1 vs 3.3, IQR 2.3 – 4.1 AU/pg; p < 0.0001). The AGE/sRAGE ratio positively correlated with age in controls (r=0.286, p<0.01), while in patients with HT it correlated with BMI (r=0.275, p=0.02), TSH (r=0.255, p=0.03), and inversely with fT3 (r= - 0.239, p=0.04).

## 4 Discussion

Hypothyroidism is a commonly encountered clinical condition, and a predisposing factor for cardiovascular disease, obesity and diabetes mellitus (22–24). In glucometabolic diseases hypothyroidism further worsens clinical complications *via* complex mechanisms (25), among which AGEs, together with inflammatory response might play a significant role.

Carotid intima-media thickness (cIMT) is a predictor of stroke and coronary heart disease and acts as a marker of subclinical atherosclerosis. The enhanced arterial stiffness, together with hyperlipidemia, hypercoagulable state, endothelial dysfunction caused by hypothyroidism might worsen the effect of traditional cardiovascular risk factors (26, 27). The accumulation of AGEs enhances arterial stiffness *via* structural and dynamic components. Cross-linking of AGEs on collagen and elastin makes the arterial wall stiffer, together with the glycation-provoked increased collagen and decreased elastin amount (28), while dynamic components as reduced nitric oxide (NO), increased endothelin-1 and altered neuroendocrine signaling will lead to endothelial cell damage (29–31). In two studies, cIMT was increased among euthyroid HT female patients regardless of traditional cardiovascular risk factors. cIMT was not different between low-normal and high-normal TSH categories. However, adiposity might had influenced the findings of the study (32, 33). The effect of TSH levels in the normal range on cIMT is still controversial (34, 35). Low T3 syndrome, the consequence of non-thyroid systemic diseases seems to affect the progression of atherosclerosis and T3 may serve as an endogenous protective molecule to inhibit vascular calcification (36, 37). Furthermore, both T3 and T4 are well known to modulate physiologic immune responses through genomic and nongenomic mechanisms and support the function of the immune system (38).

AGEs are elevated among patients with hypercholesterolemia, and they seem to have a common ligand-binding site on scavenger receptor class B type I, which plays a central role in reverse cholesterol transport, with oxidized (ox-LDL) and acetyl-LDL, therefore in the presence of AGEs the cholesterol efflux from cells to HDL is inhibited, resulting in accelerated atherosclerosis in diabetes mellitus (39, 40). On the other hand, statins, due to their cholesterol-lowering effects, can increase the soluble RAGE level by inducing RAGE shedding, therefore preventing the development of RAGE-mediated pathogenesis (41), this underlines the importance of screening hyperlipidemia among patients with thyroid disorders.

Obesity is a well-known complication of untreated hypothyroidism. Thyroid dysfunction is associated with changes in body weight and composition, body temperature, total and resting energy expenditure independently of physical activity. Even slight variations of thyroid function within the normal reference range can contribute to the development of regional obesity (23). According to the data of the DanThyr study, thyroid function has the same impact on BMI as tobacco smoking and physical activity. An increase in T3/T4 ratio had been also observed with increasing BMI (42). Obesity and hypothyroidism seem to have a bidirectional relationship, as thyroid disorders can also develop secondary to obesity (7, 22, 25). Leptin affects thyroid deiodinase activities resulting in activation of T4 to T3 conversion, while the low level of T3 in hypothyroid patients leads to decreased leptin expression, further decreasing conversion of T4 to T3 (7). Besides, T3 directly stimulates food intake at the level of the hypothalamus (22, 43). A recent systematic review of 22 studies found obesity to be significantly associated with an increased risk of hypothyroidism and clearly associated with Hashimoto thyroiditis (8). In obesity, the thyroid hormone transport through cell membrane becomes impaired due to the low-grade inflammation leading to thyroid hormone resistance in obesity (44–46). As we found a negative correlation between BMI and sRAGE levels in both patient and control group, we can conclude that overweight/obese state is a risk factor for disorders based on inflammation and oxidative stress, independently of the presence of HT. The correlations we found were present in all weight cohorts (data not shown). Among both groups AGE was positively correlated with age and serum creatinine. The increment of AGEs with advanced age is well documented in the literature, as a consequence of the life-long accumulation of AGEs and the deterioration kidney function (47, 48). Besides that, in HT patients fT3 levels also correlated with age and serum creatinine levels.

It remains to clarify if the modulated AGE and RAGE patterns in our treated HT group is related to the low grade inflammation present in HT or to the thyroid hormonal environment which is different in endogenous and exogenously achieved hypothyroidisms (49).The majority of the HT patients studied presented with chemical euthyroidism with normal TSH. The fT4 and fT3 pattern of the HT group was characteristic of T4 treated hypothyroidism.

Altogether, alterations in thyroid hormone levels may favor obesity, atherosclerosis, and inflammation; besides, thyroid dysfunction contributes to the activation of inflammation and immunity response, leading to a bidirectional crosstalk between obesity and thyroid autoimmunity (38, 45). In a recent study of Ruggeri et al. 81 newly diagnosed HT patients’ and 119 controls’ dietary habits were evaluated through questionnaires. HT patients reported higher intake frequencies of animal foods, and AGEs were also higher among them. A protective effect seems to exist of low intake of animal foods toward thyroid autoimmunity with a positive influence of higher intake of plant-based nutritional patterns on redox balance and potentially on oxidative stress-related disorders (9). Among HT children a recent pilot study showed decreased sRAGE levels with no difference in AGE levels (50). In the study of Ruggeri et al. untreated Hashimoto patients were enrolled, and from their results the malevolent change in AGE/sRAGE ratio could be predicted to worsen during the course of HT leading to hypothyroidism. However, surprisingly, in our study the patients’ AGE levels were lower than the control groups’, while sRAGE levels were higher among patients (5). Further studies would be beneficial to evaluate the effect of levothyroxine supplementation on subclinical inflammation in euthyroid Hasimoto's thyroiditis population. Altogether, the association of sRAGE with BMI might imply that maintaining normal weight might be protective from the metabolic and cardiovascular complications of HT.

The results affected by lifestyle factors such as nutritional behavior and climate of different countries and geographical regions might differ and that needs to be considered when one draws consequences. The unfavorable results from our control group might be different from the Italian data due to the unhealthy Hungarian dietary habits with saturated fat consumption compared to a healthier Mediterranean diet (51). 

In a work of Giannakou et al. the increase of oxidative stress determined by total lipid peroxide levels in serum was higher among HT patients receiving LT4-supplementation; in the overweight/obesity patient group and among patients with low fruit and sporadic vegetable consumption (52). The oxidative stress was elevated among treated patients, furthermore, RAGE 429T>C polymorphism, which is a supposed risk factor for diabetic macrovascular complications and nephropathy bore also an elevated risk for developing hypothyroidism among HT patients (6, 53).

Besides their role in oxidative stress AGEs are elevated with unhealthy nutritional habits, further worsening (or originally initiating) the putative mechanism in HT pathogenesis.

Levothyroxine treatment decreases oxidative stress when initiated in overt hypothyroidism (54–56) or subclinical hypothyroidism (57). However, in long time continued LT4 therapy parameters of oxidative stress might be elevated when compared to controls (58, 59). In our study, AGEs were lower and sRAGE was higher in HT patients, further confirming the antioxidative effect of LT4 substitution in HT. We showed correlation between TPOAb and AGE level. We are the first to evaluate AGEs and RAGE levels among LT4-treated HT patients.

Elevated cIMT and the change in AGE/RAGE axis might play a role in atherosclerosis among HT patients. In our study anti-TPO levels correlated with AGE levels, but the AGEs were not higher among LT4-treated HT patients than in healthy controls, which is in concordance with the decrement of oxidative stress among subjects on LT4-substitution (54).

According to the performed multiple regression analysis, creatinine and TPOAb levels proved to be significant predictors of AGEs, while BMI and TSH were significant predictors of sRAGE (**Table 2**).

The limitations of the study are relatively low number of patients, the wide age range of the studied population, and the lack of a HT patient group without levothyroxine substitution and evaluation of oxidative and inflammatory parameters. Higher patient number with measurement of arterial stiffness and cIMT could add to our results in terms of the effect of life-long AGE accumulation regarding the cardiovascular risk factors in HT patients in a prospective longitudinal study. The strength of our study is that we are first to demonstrate a lower AGE/sRAGE ratio among treated HT patients than controls, and the negative correlation between AGE and fT3 levels, together with correlation between sRAGE and TSH levels.

Based on our results, we assume that levothyroxine supplementation may contribute to a more favorable AGE/sRAGE ratio in treated HT patients, but at the same time, patient-specific correlations suggest that higher fT3 levels among treated patient; as well as a lower TSH level within the reference range, may have a more favorable effect on oxidative stress. However, the use of combination therapy with both levothyroxine and liothyronine remains highly controversial with conflicting results from published clinical trials. Combination therapy might be beneficial for patients with persistent symptoms despite adequate doses of levothyroxine, after having closed out alternative causes originated from a concomitant disease; but not recommended for patients who are pregnant or trying to conceive, or with arrythmias or established cardiovascular disease (60, 61).

The modulation of the AGE-RAGE axis can be attained by the modification of lifestyle, cooking with moist heat, at lower temperatures, increasing physical activity and smoking cessation (12). All these might be useful tools of AGE decrement in general, and specifically in patients with Hashimoto’s thyroiditis.  

## Data availability statement

The raw data supporting the conclusions of this article will be made available by the authors, without undue reservation.

## Ethics statement

The studies involving human participants were reviewed and approved by Regional and Institutional Ethics Committee of the University of Debrecen. The patients/participants provided their written informed consent to participate in this study.

## Author contributions

Study design: EB and MB. Development of methodology: MK, HL, and SC. Collection of data: IM, SH, DH, and SS. Analysis and/or interpretation of data: MK and SC. Writing (not revising) all or sections of the manuscript: SC, EB, MB, and MH. Manuscript review: GP, EN, MB, and MH. EB and MB contributed equally to this work and share last authorship. All authors contributed to the article and approved the submitted version.
